# Changing practice in the assessment and treatment of somatosensory loss in stroke survivors: protocol for a knowledge translation study

**DOI:** 10.1186/s12913-018-2829-z

**Published:** 2018-01-23

**Authors:** Liana S. Cahill, Natasha A. Lannin, Yvonne Y. K. Mak-Yuen, Megan L. Turville, Leeanne M. Carey

**Affiliations:** 1 Occupational Therapy, School of Allied Health, La Trobe University, Bundoora, Australia; 2 Neurorehabilitation and Recovery, Stroke Division, The Florey Institute of Neuroscience and Mental Health, Melbourne, Australia; 3 School of Allied Health, Australian Catholic University, Melbourne, Australia; 4 Department of Occupational Therapy, Alfred Health, Melbourne, Australia

**Keywords:** Somatosensory disorders, Translational medical research, Clinician behavior change, Occupational therapy, Physiotherapy, Rehabilitation, Complex intervention

## Abstract

**Background:**

The treatment of somatosensory loss in the upper limb after stroke has been historically overshadowed by therapy focused on motor recovery. A double-blind randomized controlled trial has demonstrated the effectiveness of SENSe (Study of the Effectiveness of Neurorehabilitation on Sensation) therapy to retrain somatosensory discrimination after stroke. Given the acknowledged prevalence of upper limb sensory loss after stroke and the evidence-practice gap that exists in this area, effort is required to translate the published research to clinical practice. The aim of this study is to determine whether evidence-based knowledge translation strategies change the practice of occupational therapists and physiotherapists in the assessment and treatment of sensory loss of the upper limb after stroke to improve patient outcomes.

**Method/design:**

A pragmatic, before-after study design involving eight (*n* = 8) Australian health organizations, specifically sub-acute and community rehabilitation facilities. Stroke survivors (*n* = 144) and occupational therapists and physiotherapists (~10 per site, ~*n* = 80) will be involved in the study. Stroke survivors will be provided with SENSe therapy or usual care. Occupational therapists and physiotherapists will be provided with a multi-component approach to knowledge translation including i) tailoring of the implementation intervention to site-specific barriers and enablers, ii) interactive group training workshops, iii) establishing and fostering champion therapists and iv) provision of written educational materials and online resources. Outcome measures for occupational therapists and physiotherapists will be pre- and post-implementation questionnaires and audits of medical records. The primary outcome for stroke survivors will be change in upper limb somatosensory function, measured using a standardized composite measure.

**Discussion:**

This study will provide evidence and a template for knowledge translation in clinical, organizational and policy contexts in stroke rehabilitation.

**Trial registration:**

Australian New Zealand Clinical Trials Registry (ANZCTR) retrospective registration ACTRN12615000933550.

**Electronic supplementary material:**

The online version of this article (10.1186/s12913-018-2829-z) contains supplementary material, which is available to authorized users.

## Background

Upper limb somatosensory loss after stroke is common, with over half of stroke survivors affected [1–5]. Occupational therapists and physiotherapists play a crucial role in the assessment and treatment of somatosensory loss after stroke. Despite the prevalence of somatosensory impairment, clinicians and researchers have historically given precedence to the motor sequelae of stroke, neglecting somatosensory rehabilitation [6]. The evidence-practice gap in somatosensory assessment and treatment after stroke was highlighted in a cross-sectional study of 172 occupational therapists and physiotherapists practicing in Australia [7]. Results revealed the majority (>90%) viewed assessment and treatment of sensory loss after stroke as important, however over 70% did not use standardized assessments and 33% used no specific approach to treatment. Similar findings came from the United States, where practice patterns of 145 occupational therapists were studied; 93% of those surveyed regularly assessed upper limb somatosensory function, though only half reported always or frequently providing interventions to target upper limb somatosensory loss [8].

Somatosensation involves the detection, discrimination and recognition of body (somato) sensations such as touch, vibration, temperature, proprioception and pain [9]. Impairments of somatosensation vary in severity. Moreover, these somatosensory impairments have been associated with impaired grasp and manipulation of objects [10] and poorer functional outcomes [11, 12]. The wide-ranging impact of somatosensory loss necessitates the use of sensitive assessment measures and effective, evidence-based treatment approaches. Unfortunately, the assessment and treatment of these deficits are often not addressed, or addressed inadequately, in clinical settings which often leads to poor outcomes for stroke survivors.

The evidence base for remediation of somatosensory impairment after stroke has expanded in recent years with the publication of high-level evidence. SENSe (Study of the Effectiveness of Neurorehabilitation on Sensation) therapy retrains sensory discrimination in the upper limb and targets three aspects of somatosensation: texture discrimination, limb position sense and tactile object recognition [13]. In contrast to early methods of sensory rehabilitation involving bombardment (i.e. rubbing or icing the affected limb or immersing the affected hand in containers of rice or sand), SENSe therapy capitalizes on neural plasticity and uses perceptual learning and theories of recovery following brain injury [9]. The therapy uses purpose-designed equipment such as texture grids, a proprioceptive apparatus with forearm and hand splints and everyday objects for haptic recognition (see Additional file 1). SENSe therapy was studied in a double-blind randomized controlled trial involving 50 stroke survivors. The control group received ten, 1-h therapy sessions of repeated, non-specific exposure to sensory stimuli via passive movements and grasping common objects, while the intervention group received ten, 1-h therapy sessions involving SENSe therapy. Results demonstrated significantly greater improvement in functional sensory discrimination capacity in the intervention group (t(47) = 2.75, *p* = .004, 1-tailed; effect size d = 0.79), with improvements maintained at 6 weeks and 6 months [13]. Sensory discrimination training involving SENSe therapy is now recommended in clinical practice guidelines for stroke [14].

Clinical practice guidelines are a useful tool to guide knowledge translation. However, a systematic review of the use of clinical guidelines revealed low adoption and adherence rates, even when high awareness and agreement with guidelines were reported [15]. It is proposed clinical guidelines are combined with active knowledge translation interventions to increase the likelihood of their integrated and sustained use in stroke care [16, 17].

Knowledge translation, also known as implementation, is a dynamic process of moving knowledge to practice and is based on the growing movement of implementation science [18]. Strategies for knowledge translation aim to elicit change in healthcare organizations, the behavior of healthcare professionals or the use of health services by healthcare recipients [19]. To date, there has been limited knowledge translation research in stroke rehabilitation and information is required to support clinicians, health organizations and policy makers direct finite health resources to improve patient care. Rehabilitation therapies often involve a complex integration of knowledge and skill and just as this poses particular challenges in undertaking randomized controlled trials, it also poses these same challenges when seeking to implement research findings into practice.

In this paper we describe the study protocol for a project designed to address the knowledge-practice gap in delivery of an effective upper-limb neurorehabilitation therapy - *Translating neurorehabilitation research into clinical practice: The SENSe Implement project*.

### Theoretical framework

The importance of using a guiding theoretical framework for knowledge translation efforts is now widely accepted and advocated [20, 21]. Theories proposed are usually aimed at behavior change and these frameworks provide a means of categorising interventions and identifying possible mechanisms to affect change [22]. This current study will be guided by the Theoretical Domains Framework (TDF) [23, 24]. The TDF integrates multiple theories and key theoretical constructs related to behavior change and synthesizes these into a single framework to assess and guide translational activities. Researchers can consider the 14 domains outlined in this framework, for example: knowledge, beliefs about capabilities, optimism, environmental context and resources and emotions, to explore potential barriers and enablers for knowledge translation and formulate interventions. To support the design of knowledge translation strategies for this study, the Behavior Change Wheel will be used to identify the target behavior required in terms of capability, opportunity and motivation [25]. To avoid a ‘theoretical straightjacket’ in this study, guidance will also be sought from Normalization Process Theory [26, 27] to consider the determinants of embedding (that is, normalizing) complex interventions in practice.

### Study aims

The overall aim of this study is to improve occupational therapists’ and physiotherapists’ use of research evidence to narrow the evidence-practice gap and improve the health outcomes of those who experience upper limb somatosensory loss after stroke. Component aims of this study are to investigate the effect of using multi-component evidence-based knowledge translation strategies to change the assessment and treatment practices of occupational therapists and physiotherapists working with stroke survivors with upper limb sensory loss. Additionally, this study will establish and disseminate a template of how high-level evidence in stroke rehabilitation may be systematically implemented in clinical settings using a theoretical framework for knowledge translation.

It is hypothesized that:

i) Occupational therapists and physiotherapists trained in standardized somatosensory assessment measures and the evidence-based SENSe intervention will report increased knowledge and skill in the assessment and treatment of impaired texture discrimination, limb position sense and tactile object recognition of the upper limb in stroke survivors.
ii) The use of systematic and evidence-based knowledge translation methods will be associated with an increase in reported confidence and use of evidence-based approaches for sensory rehabilitation post-stroke.
iii) Adult stroke survivors who receive SENSe therapy will have improved somatosensory capacity and hand function compared to adult stroke survivors receiving usual care.

## Methods

### Study design

This study will use a before-after design in eight Australian healthcare organizations where occupational therapists and physiotherapists provide rehabilitation to stroke survivors (see http://www.anzctr.org.au for study sites). The study has two phases:

#### Phase one: Usual care

In this phase, therapists will provide usual care to stroke survivors with somatosensory loss. Usual care is defined as the treatment therapists normally provide to stroke survivors and is likely to have natural variations depending on the healthcare organization. In the context of this study, usual care will be interpreted with reference to routine somatosensory assessment and treatment as reported in a cross-sectional study of current practices in Australia [7].

#### Phase two: SENSe therapy

In this phase, participating therapists will be up-skilled in evidence-based treatment approaches for somatosensory loss after stroke (SENSe therapy) and provide this therapy to stroke survivors. Therapists will use principles of sensory discrimination training to address texture discrimination, limb position sense and tactile object recognition. In addition, these principles will be applied in the context of functional upper limb tasks identified by the stroke survivors as being impacted by their somatosensory impairment. Treatment sessions are conducted on a 1:1 basis and individually tailored. They will involve approximately 10 sessions of 1-h duration across several weeks, with variations depending on the nature of service delivery at participating sites.

### Participants and recruitment

This study will involve two participant groups:

1. *Occupational therapists and physiotherapists*:

Qualified occupational therapists and physiotherapists will be eligible for recruitment if they work in a clinical setting with stroke survivors in a public or private healthcare organization. Therapists will be purposively recruited from organizations in both metropolitan and regional areas of Australia.

Recruitment of occupational therapists and physiotherapists will occur through an information presentation held at their workplace, conducted by an associate researcher in the study. Details of the study will be provided and therapists will decide whether they would like to participate. Participant information and consent forms will be provided at this time.

2) *Stroke survivors*:

Individuals with a recent or past history of ischemic or hemorrhagic stroke who are presenting with upper limb somatosensory loss (impaired touch discrimination, limb position sense and/or tactile object recognition) will be eligible for recruitment. Recruited stroke survivors will be aged over 18 years, medically stable, able to give informed consent and able to follow multiple-staged commands. Stroke survivors will be excluded from the study if they present with marked unilateral spatial neglect, have a past history of other central nervous system dysfunction or have peripheral neuropathy affecting their upper limbs.

Eligible stroke survivors will be identified by participating occupational therapists and physiotherapists. Consecutive sampling will be used to recruit stroke clients from both inpatient and community-based settings.

#### Sample size

A power calculation was used to determine the number of stroke survivor participants needed to achieve study objectives. An effect size of d = 0.79 was observed in the randomised controlled trial of SENSe therapy [13]. Allowing that variation in therapist experience and adaptation to local context may result in a reduced effect size, the sample size selected is aimed at detecting an effect that is 0.6 of the SENSe Cohen d. A sample of *n* = 144 is needed to detect this effect with 80% power. The projected number of allied health professional participants (~*n* = 80) will account for anticipated workforce changes and staff attrition; up to 12 therapists will be recruited at each site.

### Knowledge translation intervention

#### Participant group 1 (occupational therapists and physiotherapists)

The multi-component knowledge translation strategies will involve:

i) *Educational meetings* [28]

Phase 1: Occupational therapists and physiotherapists will be upskilled in assessment approaches (SENSe Assess©) in workshops of 5 h, typically spread over 3 sessions across 3 weeks.

Phase 2: Occupational therapists and physiotherapists will be upskilled in treatment approaches (SENSe therapy) in workshops of 8 h, typically spread over 3 sessions across 3 weeks.

ii) *Educational materials* [29]

Participating occupational therapists and physiotherapists will have access to resources and guidelines for the appropriate setup of equipment and recording assessment and treatment results.

iii) Provision of equipment for assessment and treatment

Participating organizations will be provided with the equipment necessary to conduct standardized assessments and deliver SENSe therapy.

iv) *Educational outreach visits* [30]

Associate researchers will visit participating sites to provide information and support to guide knowledge translation.

v) *Local opinion leaders* [31]

Establishment and fostering of ‘champion therapists’ will occur at each site to influence and encourage knowledge translation.

vi) *Audit and Feedback* [32]

Associate researchers will compile summary information from medical records regarding components of completed assessment and treatment and provide this to participating occupational therapists and physiotherapists with recommendations for future clinical practice.

Additionally, interventions to change practice will be tailored to individual sites through the use of pre-implementation questionnaires and focus groups regarding barriers and enablers. These currently undetermined factors will be mapped to potential interventions using the behavior change wheel [25].

### Primary outcomes

#### Participant group 1 (occupational therapists and physiotherapists)

Change in practices of participating therapists (*n* = ~80) will be evaluated by:

1. *Pre- and post- knowledge translation questionnaires*

Therapists’ knowledge, perceived skill, confidence levels and use of assessment and treatment approaches for post-stroke somatosensory loss will be surveyed before and after intervention phases using specially designed questionnaires. Questionnaires designed for this study have been formulated based on: i) evidence from literature on somatosensory assessment and treatment, ii) a review of surveys in stroke research and iii) the domains of the Theoretical Domains Framework [24]. Questionnaires involve a combination of dichotomized, Likert-type scale categories, frequency ratings and multiple response options together with open-ended written responses.

Questionnaire data will be collected in hard copy from on-site visits by researchers, de-identified, scanned and stored securely in hardcopy and electronic form.

2. *Focus groups*

Semi-structured focus group interview questions have been designed based on the Theoretical Domains Framework (TDF) [24] and Normalisation Process Theory [26]. Pre-implementation focus group questions consider current practice regarding the assessment and treatment of sensory loss after stroke and site-specific barriers and enablers for knowledge translation. Questions are designed to provide insight into: beliefs about capacities; skills; optimism; environmental context and resources; social influences; social professional role and identity; emotion; beliefs about consequences; reinforcement; motivation and goals [24]. The purpose of the post-implementation questions is to elicit group perspectives and themes on the implementation process and changes in professional practice of therapists. Focus groups have been included to provide qualitative insights to complement quantitative questionnaire data and to explore domains of the TDF best investigated through discussion, for example the domain of ‘Emotion’. Focus group discussion will be audio-recorded for later transcription. The data will be de-identified and stored electronically in secure, password-protected files.

3. *Audits of medical histories*

An audit checklist of medical histories and treatment notes has been specifically designed for this study to review therapists’ use of standardized assessments and SENSe therapy. Adherence to recommended practice as outlined in assessment and training manuals and procedures will also be evaluated.

Clinician implementation questionnaires will be conducted at baseline, that is, the beginning of phase one (usual care), following recruitment of half of the site-specific nominated sample (end of phase one), and after the period of knowledge translation of SENSe therapy (phase two). Focus groups will occur at baseline and at the end of phase two. Audit of histories will occur during phase one and phase two.

#### Participant group 2 (stroke survivors)

Change in upper limb somatosensory function pre-post usual care or SENSe therapy will be measured using the SENSe Assess© tool [13, 33], completed by participating occupational therapists and physiotherapists following specialized training. SENSe Assess© is a composite measure of functional somatosensory discrimination capacity derived from standardized measures of texture discrimination, limb position sense and tactile object recognition. A composite measure has been selected as sensation is trained across multiple modalities. This approach is consistent with the primary outcome used in the SENSe randomized controlled trial [13]. Tests contributing to this composite measure of sensation include:

1. *Tactile Discrimination Test (TDT)* [34]

The TDT is a quantitative measure of ability to discriminate differences in finely graded texture surfaces using a three-alternative, forced choice design. The patient is asked to tactually explore the sets of texture grids with their preferred finger and indicate the one that is different.

2. *Wrist Position Sense Test (WPST)* [35]

The WPST is a quantitative measure of an individual’s capacity to determine their wrist position with vision occluded, while an examiner imposes movements at the wrist. Position sense is indicated in degree angle by the patient moving a lever with their unaffected hand to indicate wrist position.

3. *Functional Tactile Object Recognition Test (fTORT)* [36]

The fTORT measures the recognition of everyday objects through a sense of touch. Patients are presented with items (for example, a house key or watch) with different sensory attributes and asked to determine what they are feeling with vision occluded.

Each of the above component measures has age-adjusted normative standards, high reliability (*r* = 0.85 to 0.92) and good discriminative test properties [33–35]. Criterion of abnormality has been established for each test.

The SENSe Assess© measure will be conducted for each stroke survivor at baseline and after a period of usual-care control intervention (phase one) and at baseline and after SENSe therapy intervention (phase two). The stroke survivors involved in phase one will be different to the cohort involved in phase two of the study.

### Secondary Outcomes

The following outcome measures will also be used with participating stroke survivors:

1. *The Hand Function Survey (HFS)* [37]

The HFS is a questionnaire designed to measure self-reported ability to use the affected hand during 13 everyday tasks in people with stroke. The HFS has established psychometric properties and is practical for clinical use.

2. *The Jebsen Taylor Hand Function Test (JTHFT)* [38]

The JTHFT has been widely used, has favorable psychometric properties and has normative scores for age, gender and hand dominance. The two items of the JTHFT selected for this study involve a pinch grip action.

The HFS and JTHFT will be conducted at baseline and after a period of usual care control intervention or SENSe therapy.

3. *The Canadian Occupational Performance Measure (COPM)* [39].

The COPM will be used to measure a stroke survivor’s individually identified problem areas in daily function related to sensory impairments of the upper limb. The importance attributed to the activities by the stroke survivor and the associated performance and satisfaction scores will be obtained. This measure is used to inform activities used in SENSe therapy. The COPM will be conducted at baseline and after SENSe therapy intervention in phase two only.

### Fidelity of treatment

Therapists will be required to meet knowledge and skill-based criterion during training before being able to implement SENSe therapy. The number and content of treatment sessions for each stroke participant will be monitored via specially designed training forms, completed by participating therapists. A comparison will be made with the therapy provided in the original randomized controlled trial using SENSe therapy [13] involving a checklist to evaluate treatment fidelity.

### Statistical analyses

Quantitative pre-post knowledge translation questionnaire data will be summarized in terms of occupational therapists’ and physiotherapists’ change in knowledge, reported skill and confidence, and use of trained approaches. Responses from questionnaires will be analyzed for response tendencies using contingency tables and chi-square analysis. Information will be graphically represented in relation to frequency, central tendency and variance.

Planned comparisons of pre-post sensory intervention data will contrast: i) the reduction in standardized sensory deficit score on the SENSe Assess© measure during ‘usual care’ and SENSe rehabilitation phases (group effect of SENSe intervention across sites and patients) and ii) reduction in SENSe Assess© scores during ‘usual care’ compared to SENSe intervention at each site (clinic effect). In addition, magnitude of change in SENSe Assess© scores following the phase of SENSe therapy will be benchmarked with change scores obtained in the original SENSe randomized controlled trial [13] with significance being considered using 95% confidence intervals.

Qualitative focus group data will be analysed using directed content analysis [40] with the assistance of a qualitative software package (NVivo10). Directed content analysis will allow the use of an existing theoretical framework (the Theoretical Domains Framework) to guide initial coding categories. A coding schedule will be developed, with two authors independently reviewing and coding transcripts. Discrepancies or disagreements will be resolved via consultation with a third author. Trustworthiness of data will be addressed through the documentation of characteristics of participants, data triangulation (with comparison of open-ended questionnaire and focus group information) and researcher triangulation in analysis and review of field notes. Member checking will also occur via provision of a written summary of discussion to a research associate at each site, for comment regarding accuracy. An audit trail will be kept during the analysis process.

### Study reporting

The reporting of the results of this study will be guided by the TREND statement for nonrandomized evaluations of behavioral and public health interventions [41] and the recently released Standards for Reporting Implementation Studies (STaRI) [42] statement.

### Progress to date

The study has commenced at eight sites, with pre-implementation questionnaire data collected and focus groups conducted. Occupational therapists and physiotherapists at these sites have been trained in quantitative assessment approaches for phase one of the study and have commenced the recruitment of stroke survivors.

## Discussion and conclusions

Changing behavior in healthcare has well recognized complexities. Knowledge translation interventions offer health professionals, policymakers and organizations a means of identifying, synthesizing and applying research-informed knowledge to improve a healthcare system [43]. Despite the growing interest in knowledge translation processes, there is still much to be learned about the use of specific strategies to effect change in health professionals' behavior.

Health professionals working with stroke patients have an ethical obligation to provide effective care, however stroke survivors who experience somatosensory loss have been negatively affected by the evidence-practice gap in this area. An evidence-based therapy known as SENSe [13] is now available for implementation in clinical practice settings. This therapy has been systematically developed in line with the framework for development of complex interventions [17, 44]. The next step, as indicated by our survey of the evidence-practice gap [7], is to facilitate implementation into clinical practice settings.

The current study will examine the impact of knowledge translation strategies on occupational therapist and physiotherapist knowledge of evidence and practice behaviors, and further, the impact on patient outcomes. In addition to improving the outcomes for the majority of stroke survivors that experience somatosensory loss after stroke, this study has the potential to provide evidence and a pragmatic template for the knowledge translation of research in clinical, organizational and policy contexts in stroke rehabilitation.

## Additional file

Additional file 1: SENSe therapy equipment. (PDF 247 kb)

## Abbreviations

COPM: the Canadian Occupational Performance Measure.
fTORT: functional Tactile Object Recognition Test
HFS: Hand Function Survey
JTHFT: Jebsen Taylor Hand Function Test
SENSe: Study of the Effectiveness of Neurorehabilitation on Sensation
TDT: Tactile Discrimination Test
WPST: Wrist Position Sense Test
