# Supplementary material for: Changing practice in the assessment and treatment of somatosensory loss in stroke survivors: protocol for a knowledge translation study
Source: BMC Health Serv Res. 2018 Jan 23;18:34. doi: 10.1186/s12913-018-2829-z (PMC5781331; doi:10.1186/s12913-018-2829-z)
Supplement: Additional file 1: — SENSe therapy equipment. (PDF 247 kb) [file 12913_2018_2829_MOESM1_ESM.pdf]

Additional File 1: SENSe Therapy Equipment (L-R: texture grids, proprioceptive box and tactile object recognition poster)

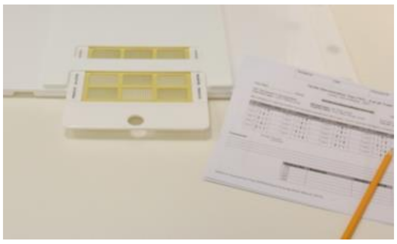

Tactile Discrimination Test
(Carey, Oke, & Matyas, 1997)

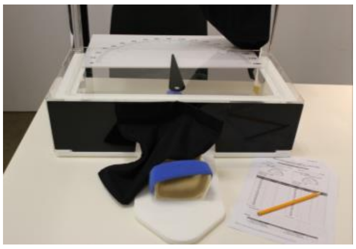

Wrist Position Sense Test
(Carey, Oke, & Matyas, 1996)

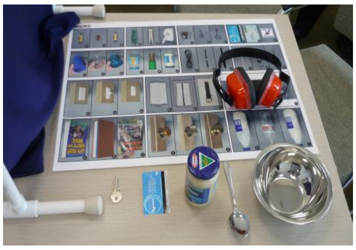

Functional Tactile Object Recognition Test
(Carey, Nankervis, LeBlanc, & Harvey, 2006)
